# The triangular fibrocartilage complex (TFCC): updated anatomy, variants, and MRI diagnostic pitfalls

**DOI:** 10.1007/s00256-026-05219-x

**Published:** 2026-04-20

**Authors:** Hicham Bouredoucen

**Affiliations:** Division of Radiology, Department of Imaging and Medical Informatics, Geneva University Hospitals, University of Geneva, Rue Gabrielle-Perret-Gentil 4, 1211 Geneva 14, Switzerland

**Keywords:** TFCC, Distal radioulnar joint, Distal radioulnar ligaments, MRI, Anatomical variants, Radioulnocarpal joint stability

## Abstract

The triangular fibrocartilage complex (TFCC) is a key stabilizer of the ulnar wrist, integrating both the distal radioulnar and ulnocarpal joints. Recent advances in arthroscopic anatomy have led to a three-dimensional, tripartite conceptualization of the TFCC, describing it as comprising three interconnected components: the articular disc, the distal radioulnar ligaments providing primary stability, and a continuous peripheral capsular structure, or “peripheral wall,” which encompasses the palmar and dorsal ulnocarpal ligaments, the meniscus homologue, and the extensor carpi ulnaris tendon sheath. Vascular supply is concentrated at the peripheral insertions, while the central disc is relatively avascular, influencing its healing potential. Functionally, the TFCC contributes to both rotational and translational stability of the radioulnocarpal articulation, with the distal radioulnar ligaments serving as the primary stabilizers and the ulnocarpal ligament complex, extensor carpi ulnaris tendon sheath, and interosseous membrane providing secondary support. MRI, particularly three-dimensional isotropic sequences and MR arthrography, allows detailed visualization of the TFCC components, detection of partial or complete tears, and differentiation between normal anatomic variants and pathologic abnormalities. Recognition of normal variations and positional changes related to forearm rotation is essential to avoid diagnostic errors. This review integrates arthroscopic, anatomical, biomechanical, and imaging perspectives, providing a comprehensive understanding of the TFCC and its updated conceptual framework, with direct implications for clinical assessment, surgical planning, and post-treatment follow-up of ulnar wrist injuries.

## Introduction

The ulnar side of the wrist represents a functional joint complex based on the coordinated interaction between the distal radioulnar joint (DRUJ) and the ulnocarpal articulation. At the center of this stabilizing system lies the triangular fibrocartilage complex (TFCC), a key structure responsible for load transmission and rotational stability of the forearm. The biomechanics of the radioulnocarpal articulation rely on both passive stabilizers—including the radioulnar ligaments, ulnocarpal ligaments, and the interosseous membrane—and dynamic stabilizers such as the pronator and extensor muscles, particularly the extensor carpi ulnaris (ECU). TFCC injuries may compromise pronation–supination and result in pain, instability, or functional limitation.

Since its initial description, the TFCC has been recognized as the central stabilizing structure of the ulnar wrist. The Palmer classification, introduced in 1989, has long served as the reference framework for evaluating TFCC lesions, distinguishing traumatic from degenerative injuries based on topographic criteria [1]. However, advances in anatomy, biomechanics, arthroscopy, and imaging have considerably expanded the spectrum of TFCC pathology, highlighting numerous peripheral, capsuloligamentous, and ulnocarpal lesions that may contribute to ulnar-sided wrist pain and instability [2–11].

Arthroscopic developments, particularly the Atzei classification incorporating the concept of ligament reparability, have improved the distinction between the superficial and deep bundles of the distal radioulnar ligaments, especially at their foveal insertions [12–14]. More recently, Herzberg proposed a comprehensive arthroscopic concept describing the TFCC as a three-dimensional tripartite structure consisting of the articular disc, the distal radioulnar ligaments acting as stabilizing “reins,” and a continuous peripheral capsuloligamentous wall [15, 16], whose involvement may contribute to DRUJ pain and instability.

In addition, patient-specific anatomical factors, such as ulnar variance and the morphology of the sigmoid notch, influence injury patterns and therapeutic decision-making [17, 18]. Advances in imaging, particularly high-resolution magnetic resonance imaging (MRI) and MR arthrography, have improved TFCC assessment, although a substantial proportion of lesions remain difficult to classify using traditional systems [19–21].

The aim of this review is to provide an integrated update of TFCC anatomy and its imaging correlations, with particular emphasis on anatomic variants and MRI diagnostic pitfalls, in order to improve diagnostic accuracy and facilitate communication with hand surgeons in the management of ulnar-sided wrist pain and DRUJ instability.

## Anatomy and structural variations of the TFCC

**Fig. 1:**
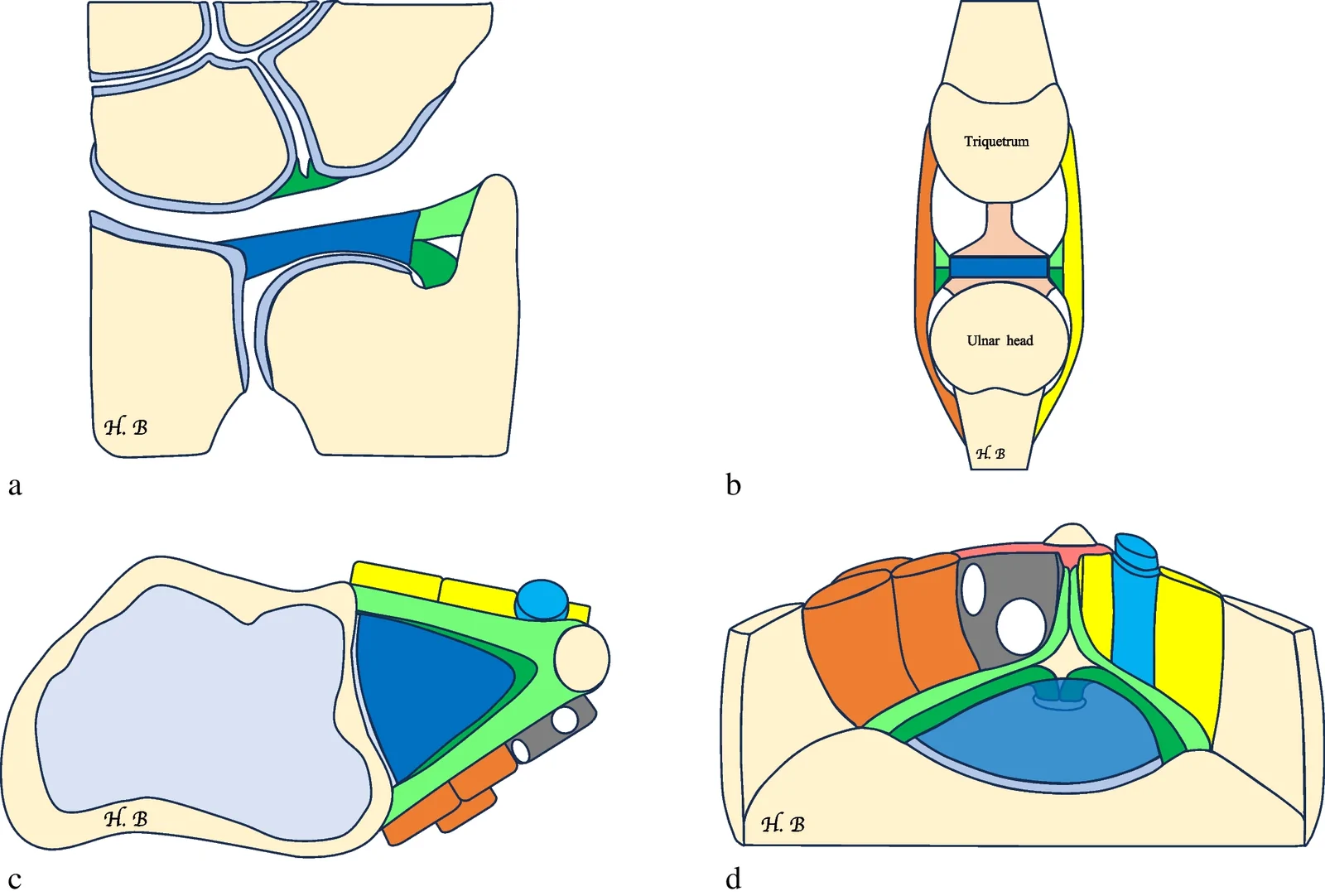
Components of the triangular fibrocartilage complex (TFCC). Schematic illustrations: coronal view (**a**), sagittal view (**b**), axial view (**c**), arthroscopic view from the 3–4 portal (**d**; dorsal entry point located between the third and fourth extensor compartments, between the extensor pollicis longus and the extensor digitorum communis/extensor indicis tendons, allowing direct visualization of the dorsal radiocarpal compartment and the TFCC). The TFCC is composed of the following: Triangular fibrocartilage (TFC, dark blue), also called articular disc; dorsal and palmar distal radioulnar ligaments: superficial fibers (light green arrows), deep fibers (green arrows); palmar ulnocarpal ligaments (ulnotriquetral and ulnolunate ligaments, orange); dorsal ulnotriquetral ligament (yellow); meniscus homologue (MH, gray); extensor carpi ulnaris (ECU) tendon subsheath (light blue); distal medial TFCC (MH: shock absorber and distal medial capsule) (light orange, distal in **b**); proximal medial TFCC (attachment of the triangular fibrocartilage to the ulnar fovea: stabilizer of the radioulnocarpal joint and proximal medial capsule) (light orange, proximal in **b**); Palmar and medial capsule (pink in **d**)

The TFCC is composed of the triangular fibrocartilage (articular disc), distal radioulnar ligaments, palmar ulnocarpal ligaments (ulnolunate and palmar ulnotriquetral), dorsal ulnotriquetral ligament, meniscus homologue (MH), and the subsheath of the ECU tendon (Fig. 1). The triangular fibrocartilage forms the central portion, while the term TFCC encompasses this disc and the peripheral capsuloligamentous complex.

### Triangular fibrocartilage

**Fig. 2:**
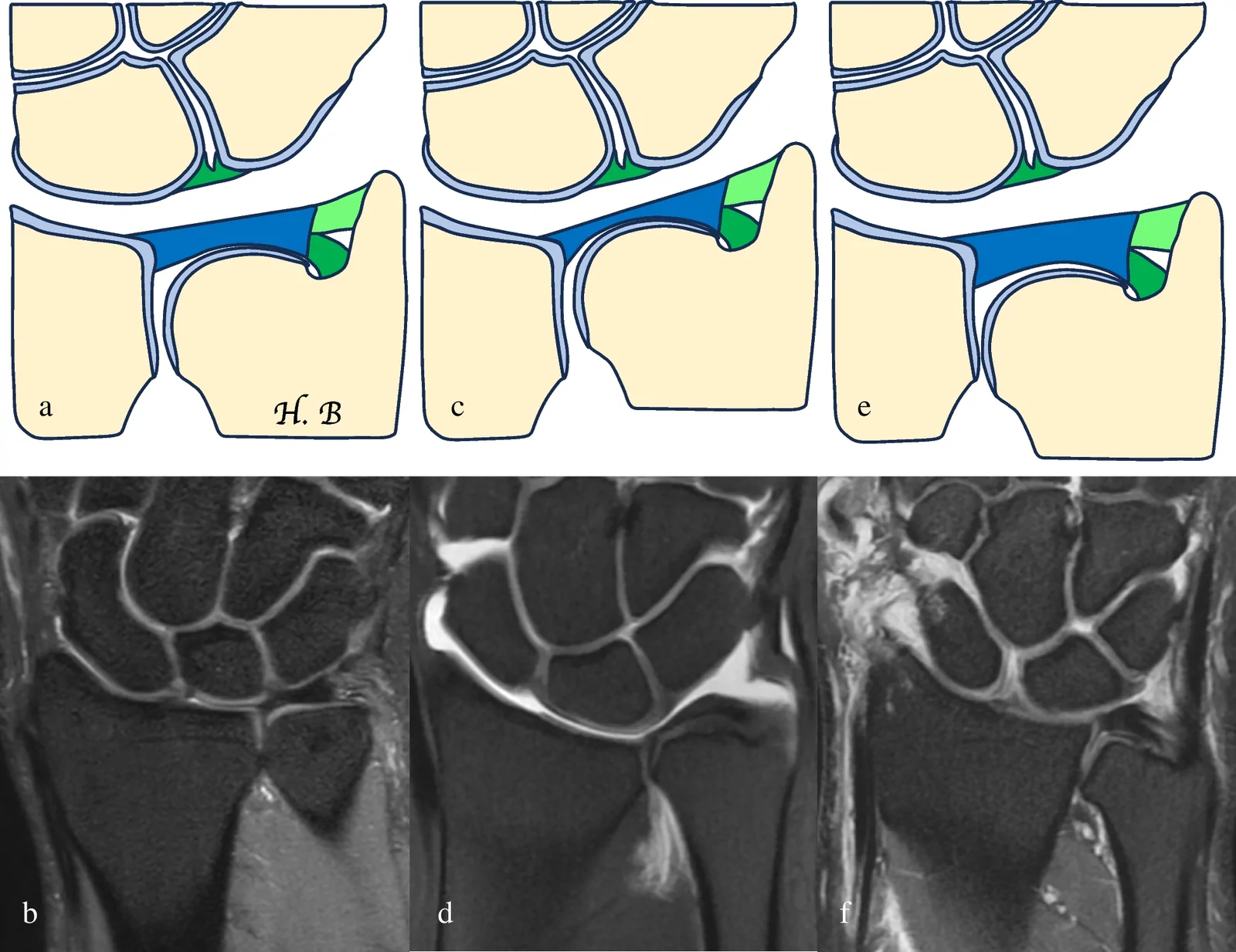
Morphology and thickness of the TFCC articular disc. Schematic illustrations (**a**, **c**, **e**) and coronal proton density fat-suppressed (PD FS) MR images (**b**, **d**, **f**). On coronal images, the disc appears thinner, more vertical, and elongated in cases of neutral (**a**, **b**) or positive (**c**, **d**) ulnar variance, whereas it is thicker and foreshortened or narrower in negative ulnar variance (**e**, **f**). This appearance in negative variance reflects the greater central thickness of the disc, which makes it visually shorter on coronal images, while the disc maintains an elongated, more vertical configuration in neutral or positive variance

**Fig. 3:**
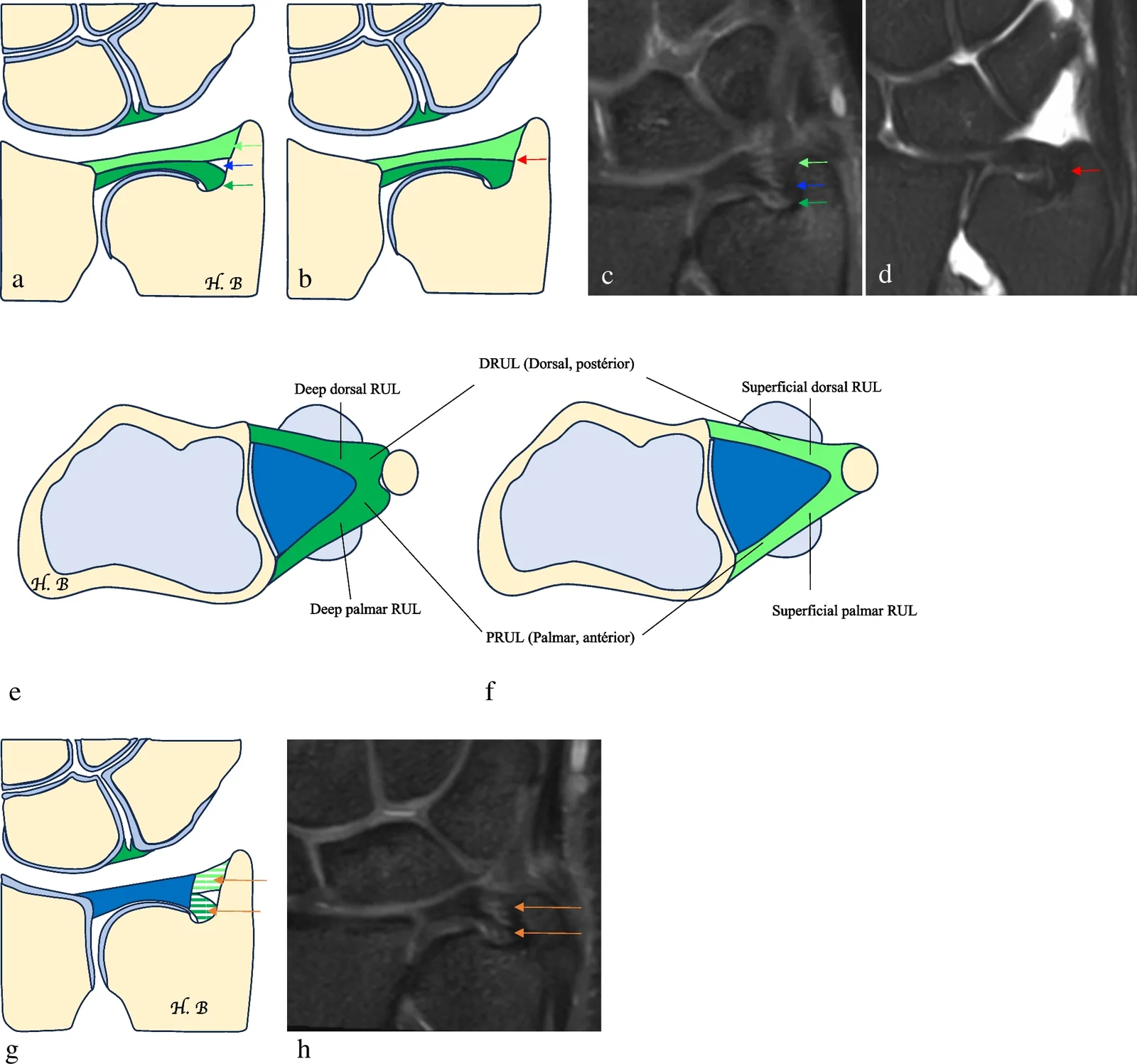
Types of ulnar attachment of the TFCC and description of the distal radioulnar ligaments. Schematic coronal views (**a**, **b**) and PD FS MR images (**c**, **d**): type 1: ulno-apical (light green arrow) and ulno-basal (green arrow) attachments (**a**, **c**). Ligamentum subcruentum (blue arrow); type 2: broad-based single attachment spanning both the ulnar fovea and the styloid, illustrating continuity between deep and superficial fibers (red arrow) (**b**, **d**). Schematic axial views (**e**, **f**). The distal radioulnar ligaments are dorsal and palmar thickenings of the articular disc. The palmar radioulnar ligament (PRUL) consists of two distinct components: a deep and a superficial portion. Similarly, the dorsal radioulnar ligament (DRUL) comprises two distinct components: a deep and a superficial portion. Schematic illustration coronal view (**g**). Sagittal view PD FS MR image (**h**). Radioulnar ligaments exhibit low signal intensity on MRI, often with a striated appearance (orange arrows)

**Fig. 4:**
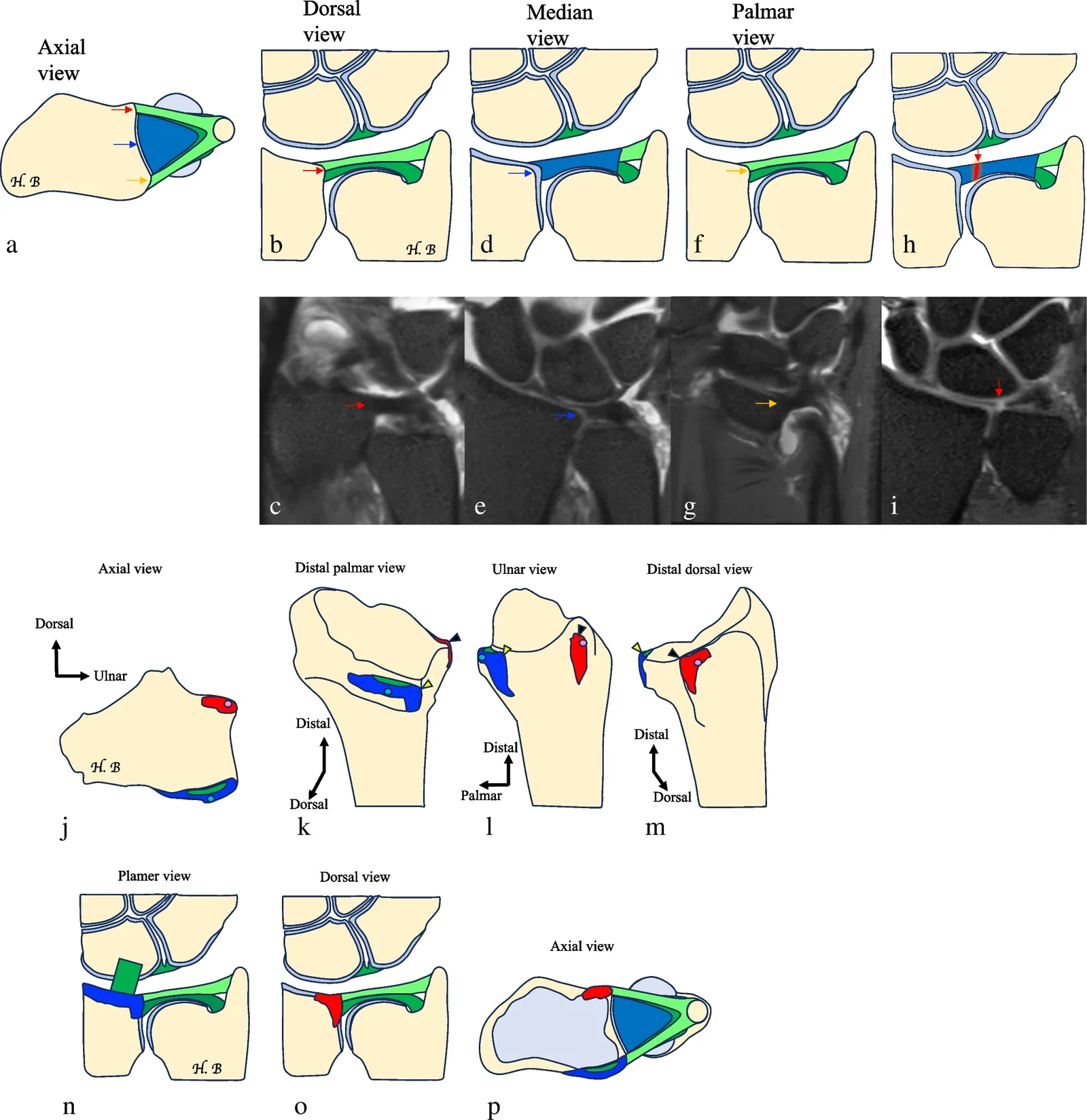
Radial attachment of the TFCC (**a**–**g**):Schematic illustrations (**a**, **b**, **d**, **f**) and coronal PD FS MR images (**c**, **e**, **g**): dorsal and volar portions attach via distal radioulnar ligaments (red and orange arrows); central portion inserts into distal radius hyaline cartilage (blue arrow). There are two types of radial tear: Tear of the articular disc attachment on the hyaline cartilage (schematic (**h**) and coronal PD FS view (**i**), red arrows), located between the palmar and dorsal ligament insertions, with intense signal adjacent to the intermediate cartilage signal; tear of the radial insertions of the distal radioulnar ligaments (palmar or dorsal), presenting an intense signal localized on the ligament insertions. Key point: The normal intermediate signal of the radial hyaline cartilage can be mistaken for a tear (false positive). A true tear of the ligament insertions presents as an intense signal localized at the palmar or dorsal insertions, while the articular disc attachment, located between these insertions, maintains its normal intermediate signal. Signal topography and intensity allow differentiation between a normal cartilaginous attachment and a ligament insertion lesion, thus avoiding false positives. Schematic illustrations of radial attachments of the radioulnar ligaments (**j**–**p**): **j** Axial view: palmar radioulnar ligament (PRUL, blue), dorsal radioulnar ligament (DRUL, red), short radiolunate ligament (green); blue and pink dots indicate the centers of the PRUL and DRUL attachments; **k**–**m** Distal palmar, ulnar, and distal dorsal views: yellow arrowheads indicate the palmar osseous apex formed by the intersection of the palmar cortex with the radioulnar and radiocarpal articular surfaces, and black arrowheads indicate the dorsal osseous apex formed by the intersection of the dorsal cortex with the same articular surfaces (referred to as the “palmar and dorsal pyramid tips”). **n**–**p** Ligaments with attachments highlighted

The triangular fibrocartilage is a biconcave triangular band extending between the distal ulna and carpal bones, attaching to the ulnar notch of the radius and ulnar epiphysis (Fig. 1). Its central portion is thin, avascular, and prone to tears. The dorsal and palmar margins merge with the distal radioulnar ligaments (Fig. 1). On coronal images, the TFCC articular disc often appears foreshortened or narrower, in addition to being thicker, in individuals with negative ulnar variance [22]. This configuration results from the greater central thickness of the disc, which tends to visually shorten it on coronal views due to its more horizontal and compact morphology. In contrast, in cases of neutral or positive variance, the disc is thinner [22], more vertical, and elongated, giving it a more extended appearance on coronal images (Fig. 2). The ulnar insertion of the disc is dual: proximal (foveal/deep) fibers attach to the ulnar fovea, while distal (styloid/superficial) fibers insert on the ulnar styloid (Fig. 1). Two types of ulnar insertions have been described: type 1 TFCC, with two insertions (ulno-apical and ulno-basal), and type 2 TFCC, which shows a broad-based single attachment spanning both the ulnar fovea and the styloid, illustrating continuity between deep and superficial fibers (Fig. 3). Radially, the disc inserts onto the hyaline cartilage (Fig. 4a–g).

### The distal radioulnar ligaments

These ligaments form a V-shaped structure connecting the radius to the ulna, with dual ulnar insertions (foveal and styloid) separated by the ligamentum subcruentum (Fig. 3a, c). Both the palmar distal radioulnar ligament (PRUL) and dorsal distal radioulnar ligament (DRUL) comprise deep and superficial bundles (Fig. 3e, f). Deep fibers insert on the fovea; superficial fibers attach to the mid-portion and base of the ulnar styloid. DRUL fibers insert radially via calcified fibrocartilage (Fig. 4j–p), whereas PRUL fibers run horizontally. The short radiolunate ligament lies deep to the PRUL, forming a conjoint insertion [23].

### Palmar ulnocarpal ligaments

**Fig. 5:**
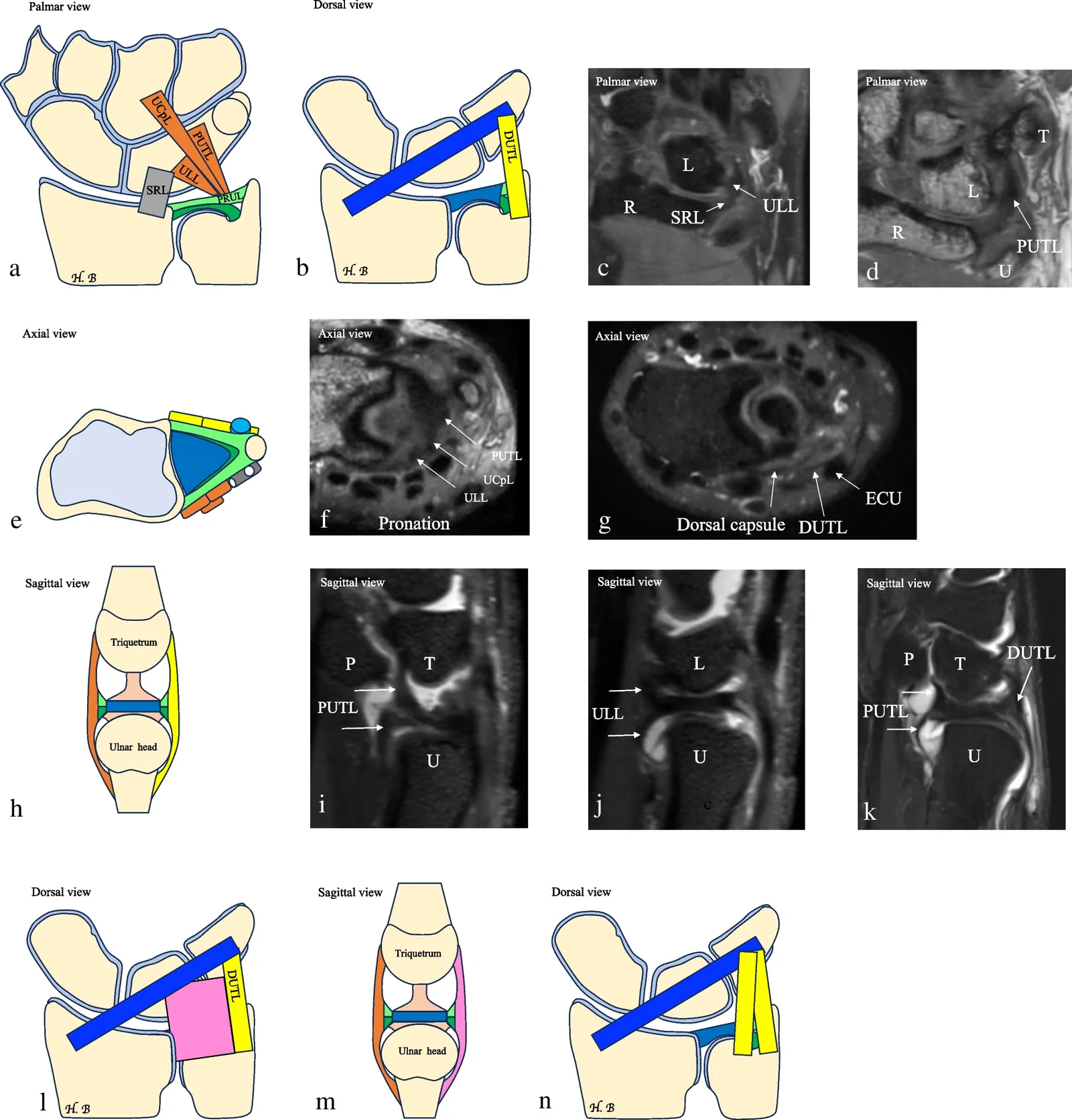
Ulnocarpal ligament complex and dorsal ulnotriquetral ligament. Schematic illustration: palmar view (**a**), dorsal view (**b**, **l**, **n**), axial view (**e**), sagittal view (**h**, **m**). PD FS MR image: coronal view (**c**), axial view (**g**), sagittal view (**i**–**k**). Proton density (PD) MR image: coronal view (**d**), axial view (**f**). Ulnocarpal ligament complex: normal anatomy, shown in schematics and MR images. The three ligaments (orange, **a**, **e**, **h**, **m**) — palmar ulnotriquetral ligament (PUTL), ulno-capitate ligament (UCpL), and ulnolunate ligament (ULL) — originate from the palmar border of the ulnar fovea and the ulnar styloid process. They fuse with the palmar distal radioulnar ligament (PRUL), deep layer (dark green, **a**), superficial layer (light green, **a**), extend distally in the coronal plane, and insert respectively on the palmar surfaces of the triquetrum, capitate, and lunate. These ligaments converge and are difficult to distinguish from one another in the absence of joint fluid or arthrographic contrast material. UCpL: does not attach directly to the TFCC but to the palmar portion of the foveal region. Fuses with the deep fibers of the PRUL at the fovea level. Passes anterior to the junction of the other two ulnocarpal ligaments and inserts on the ulno-palmar surface of the capitate. PUTL: arises primarily from the PRUL, with some fibers originating from the palmar radial surface of the ulnar styloid process. Inserts on the proximal and ulnar surfaces of the triquetrum. ULL: continuous with the short radiolunate ligament (SRL) and attaches to the palmar cortex of the lunate. Dorsal ulnotriquetral ligament (DUTL): normal anatomy and variant, represented in schematics; normal anatomy visible on MR images; extends from the ulnar styloid process to the dorsal aspect of the triquetrum (yellow, **b**, **e**, **h**); shares a broad insertion with the dorsal radiotriquetral ligament (intense blue, **b**). Trajectory perpendicular to the dorsal radioulnar ligament (DRUL), deep layer (dark green, **b**), superficial layer (light green, **b**). **l** and **m** Ulnar insertion of the DUTL (yellow), lateral dorsal capsular reinforcement (pink), dorsal radiotriquetral ligament (intense blue). **n** Unique anatomical variant of the DUTL with two ulnar insertions (yellow) and dorsal radiotriquetral ligament (intense blue). Other schematics show normal anatomy. Abbreviations: T, triquetrum; R, radius; L, lunate; U, ulna; ECU, extensor carpi ulnaris

These include the palmar ulnotriquetral, ulnolunate, and ulnocapitate ligaments (Fig. 5), originating from the fovea and ulnar styloid, merging with the PRUL, and inserting onto triquetrum, capitate, and lunate. The ulnolunate ligament significantly contributes to wrist stability [24].

### Meniscus homologue

**Fig. 6:**
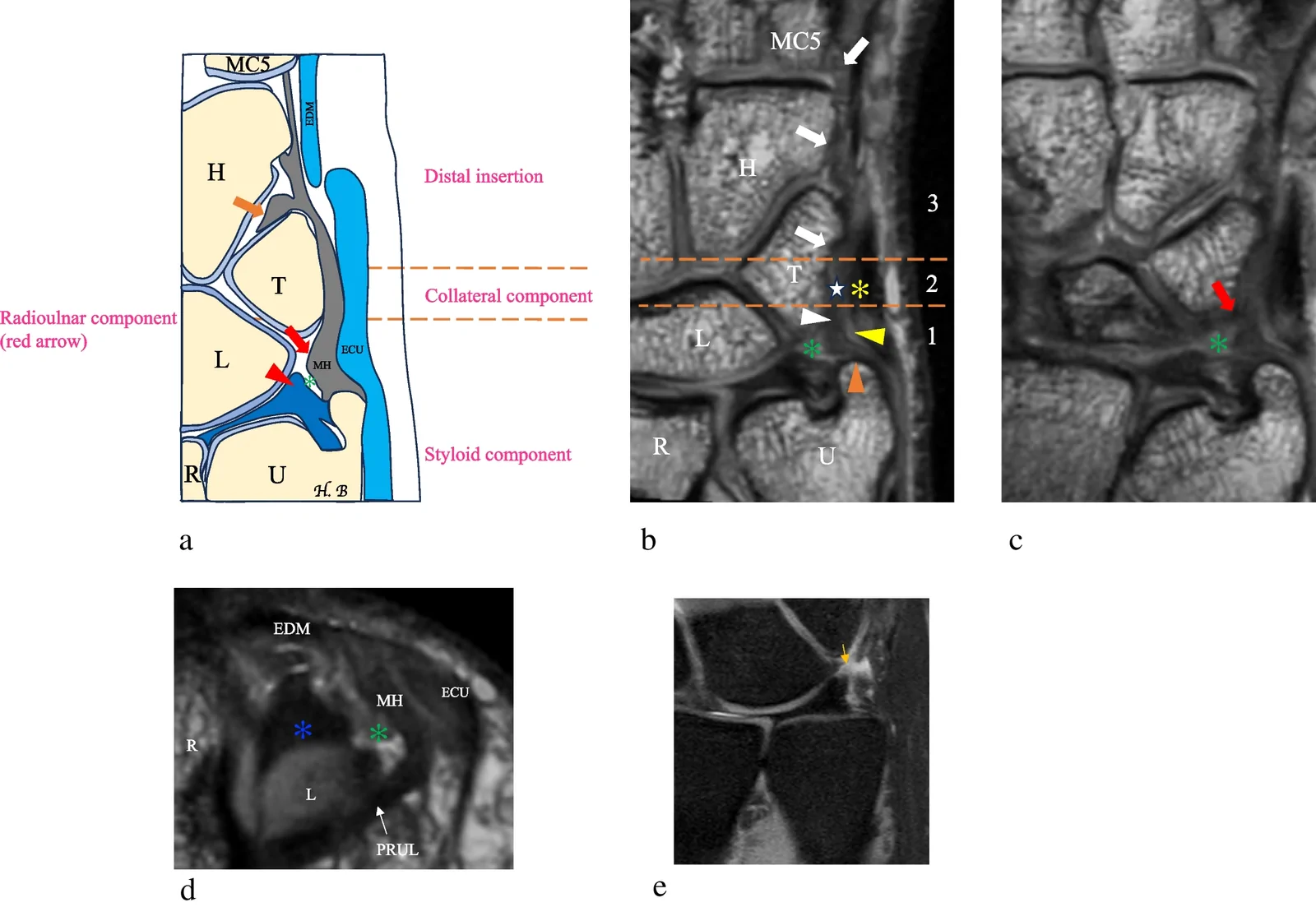
Anatomical features of the meniscus homologue (MH). **a** Schematic coronal illustration at the level of the ulnocarpal joint. The styloid component of the MH is visible at the distal tip of the ulnar styloid, comprising a ligamentous portion on the radial side and an adjacent fatty portion near the sheath of the extensor carpi ulnaris (ECU) tendon. The prestyloid recess (green asterisk, **a**) lies between the MH and the triangular fibrocartilage complex. Distally, the collateral portion of the MH and the ECU tendon sheath merge. The MH inserts distally onto the ulnar aspect of the triquetrum and the ulnar aspect of the hamate, blending with the joint capsule located between the hamate and triquetrum, forming a meniscus-like shape (orange arrow, **a**). Extensor digiti minimi (EDM). The radioulnar component of the MH (red arrow, **a**) is located near the origin of the dorsal distal radioulnar ligament (red arrowhead, **a**). **b**, **c** Coronal PD MRI images. **b** MH components: styloid component (region 1), collateral component (region 2), and distal insertion (region 3) are separated by horizontal lines. The styloid component lies between the entrance of the prestyloid recess (green asterisk, **b**) and the ECU tendon (yellow asterisk, **b**), inserting on the ulnar styloid (orange arrowhead, **b**). Its ligamentous portion is more fibrous and hypointense (white arrowhead, **b**), whereas the fatty portion is highly vascular and hyperintense (yellow arrowhead, **b**). In the collateral component, no high-intensity vascular signal is observed between the ECU tendon (yellow asterisk) and the MH (white star). The distal insertion attaches mainly to the ulnar surface of the triquetrum (T), with additional insertions on the hamate (H) and fifth metacarpal (MC5) (white arrows, **b**). **c** The radioulnar component of the MH (red arrow, **c**) lies adjacent to the prestyloid recess (green asterisk, **c**). **d** Axial PD MRI image. Prestyloid recess (green asterisk, **d**); triangular fibrocartilage (blue asterisk, **d**). **e** Prestyloid recess. Coronal PD FS MR images (**e**) demonstrating the prestyloid recess (thin orange arrow), a normal synovial pouch located between the articular disc, the meniscus homologue, and ulnar ligamentous structures, communicating with the radiocarpal joint. Its size is variable. The recess frequently contains fluid and may extend radially or ulnarly, potentially simulating a cleft within the disc or the palmar ulnotriquetral ligament. Key point: Fluid signal within the prestyloid recess can mimic a tear of the disc or the palmar ulnotriquetral ligament, representing a false-positive finding. Awareness of this anatomical variant is essential to avoid diagnostic errors. Abbreviations: H, hamate; T, triquetrum; L, lunate; R, radius; U, ulna; MC5, fifth metacarpal; MH, meniscus homologue; ECU, extensor carpi ulnaris; EDM, extensor digiti minimi; PRUL, palmar distal radioulnar ligament

On the ulnar side, the MH extends from the ECU tendon sheath to the palmar ulnotriquetral ligament, inserting on the ulnar styloid, triquetrum, hamate, and fifth metacarpal. It can be subdivided into four components: styloid, collateral, distal insertion, and radioulnar (Fig. 6). Etymologically, the MH is an evolutionary vestige from ancestral species in which the ulna directly articulated with the carpal bones, a configuration still observed in some primates. In humans, this direct articulation is mostly lost, and the MH remains as a homologue—similar in position, structure, and evolutionary origin but not necessarily in function. Tears are often associated with TFCC lesions [25, 26].

### Extensor carpi ulnaris (ECU) tendon subsheath

Dorsally, the ECU subsheath stabilizes the tendon, attaching proximally to the ulnar styloid, fovea, and DRUL, and distally to the triquetrum [27–29].

### Dorsal ulnotriquetral ligament

This ligament originates from the ulnar styloid, runs perpendicular to the DRUL, and inserts onto the dorsal triquetrum along with the dorsal radiotriquetral ligament.

### Ulnar collateral ligament of the wrist

The ulnar collateral ligament exhibits considerable variability. Historically, it has been described as distinct from the TFCC [30–34], whereas other studies report it as a thickening of the ulnar capsule in continuity with the ECU tendon sheath and MH [35–39], illustrating the challenge of defining its anatomy.

### Three-dimensional arthroscopic concept of the TFCC

Herzberg et al. [15] described the TFCC as three components: articular disc; distal radioulnar ligaments acting as stabilizing “reins”; and a continuous peripheral capsular “peripheral wall.”

### Vascularization and innervation of the TFCC

The central disc (80–85%) is relatively avascular; peripheral TFCC (15–20%) is supplied by ulnar and anterior interosseous arteries, mainly at palmar, dorsal, and ulnar attachments. Innervation arises from the posterior interosseous, dorsal and palmar ulnar cutaneous, and medial antebrachial cutaneous nerves [40]. This innervation is sensory, concentrated peripherally, and may serve as a source of pain. Accordingly, these sensory branches could represent targets for selective denervation or ablation.

## Biomechanics and stability of the radioulnocarpal joint

The DRUJ represents only one component of the complex ulnar wrist articulation. The complementary component is the ulnocarpal joint, and these two joints are functionally interdependent. To encompass the entire ulnar-sided wrist articulation, the term *radioulnocarpal joint* is therefore more appropriate. The TFCC interfaces with both the DRUJ and the ulnocarpal joint and contributes to radioulnocarpal stability during forearm rotation [41].

Radioulnocarpal joint stability is complex and depends on multiple anatomical structures [41]. Passive stabilizers include the components of the TFCC and associated ligamentous structures. The horizontal component of the TFCC comprises the central articular disc, whose primary function is to absorb axial loads transmitted across the ulnocarpal space, acting as a shock absorber with minimal intrinsic stabilizing capacity [42–44].

The distal radioulnar ligaments constitute important stabilizers of the DRUJ [24]. They play a key role in maintaining normal joint rotation when other soft-tissue stabilizers are compromised. However, when other stabilizing structures are intact, the distal radioulnar ligaments and the triangular fibrocartilage are not strictly necessary for the preservation of normal DRUJ kinematics [45].

The most common pattern of instability is dorsal displacement of the distal ulna relative to the radius [46], and the PRUL provides the greatest resistance to this translation [47]. During pronation and supination, the deep DRUL and PRUL twist around their foveal insertion [32], supporting the rationale for repair or reconstruction centered on the fovea in cases of instability.

The membrane maintaining the ECU tendon within its subsheath constitutes an important secondary stabilizer. During supination, contraction of the ECU muscle contributes to stabilization of the distal ulna [41, 44, 48].

Palmar ulnocarpal ligaments stabilize the ulnar column of the wrist and are maximally tensioned in supination. Lesions of these ligaments result in palmar displacement of the radial column relative to the ulna [41]. Radial deviation or wrist hyperextension places substantial strain on the ulnocapitate ligament and palmar ulnotriquetral ligament, as well as on the palmar foveal and styloid insertions of the TFCC [49]. Excessive traction on the ulnocarpal ligament complex—particularly the ulnocapitate ligament during radial hyperextension or supination—may result in foveal avulsion [50]. Distal fibers of the interosseous membrane, particularly the distal oblique bundle, also play a secondary stabilizing role by limiting proximal migration and dorsal translation of the radius [51, 52].

Active stabilizers of the radioulnocarpal joint are primarily muscular and contribute to articular surface coaptation. The brachialis muscle, due to its insertion on the proximal ulna, reacts to external forces transmitted from the radius during hand loading and generates the reaction force necessary to maintain DRUJ coaptation. Similarly, the abductor pollicis longus and extensor pollicis brevis, originating from the ulna and following an oblique course toward the first extensor compartment of the radius, contribute to distal radioulnar stability by compressing the DRUJ during their contraction [41].

## MRI

Optimal imaging of the TFCC requires high spatial resolution and signal-to-noise ratio, which are influenced by field of view, matrix size, echo time, slice thickness, bandwidth, repetition time, sequence selection, and magnetic field strength. Increasing the matrix or reducing the field of view improves spatial resolution [53], further enhanced by dedicated coils and a 3 T field, preferred over 1.5 T [54, 55]. Studies comparing 7 T and 3 T have demonstrated comparable performance in detecting TFCC lesions, confirming the adequacy of current 3 T systems [56, 57]. Deep learning–based reconstruction techniques can further improve image quality and reduce acquisition time, even at lower field strengths [58].

**Fig. 7:**
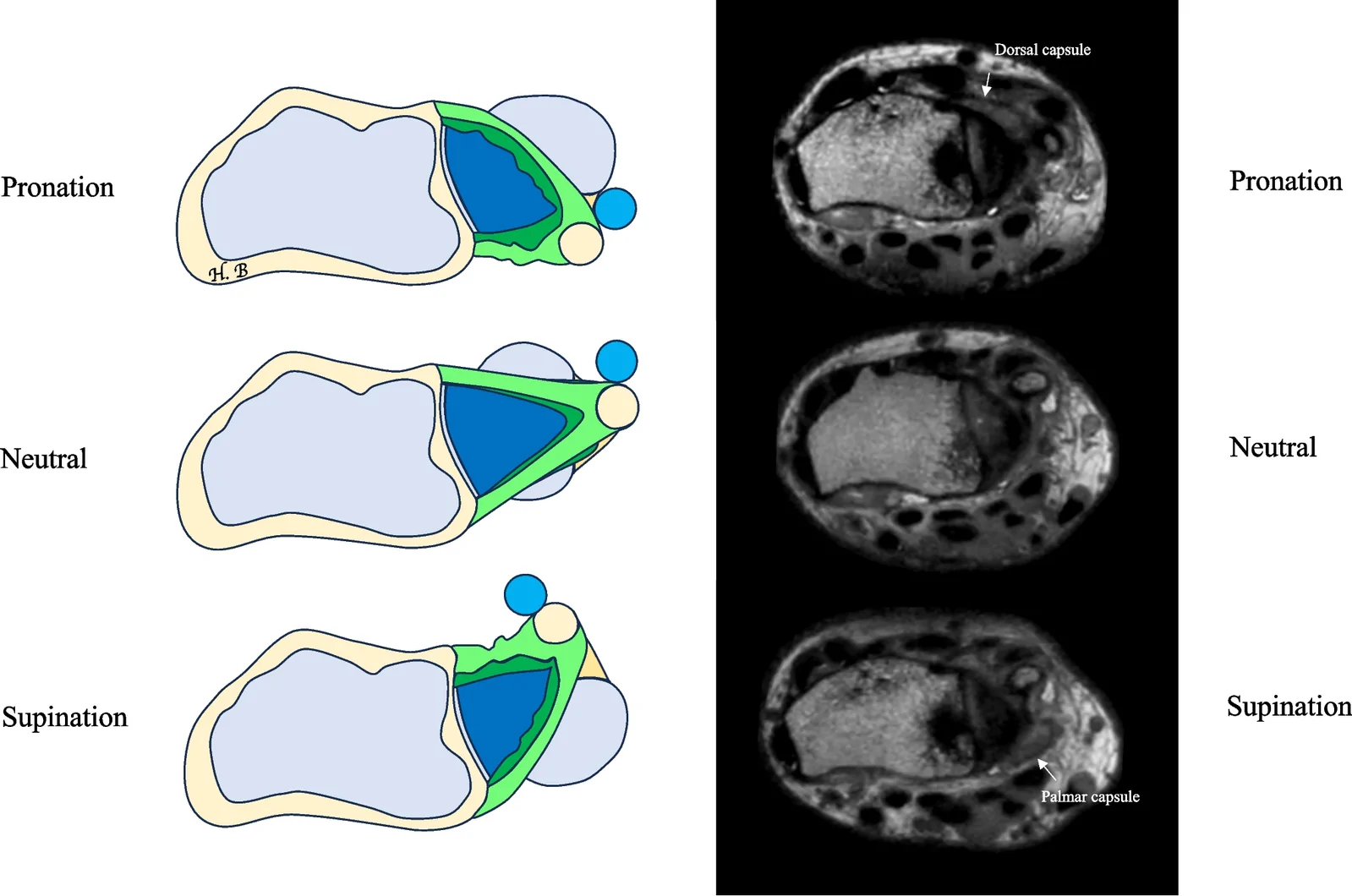
Changes in the Triangular Fibrocartilage Complex (TFCC) during pronation and supination. Schematic illustration in axial sections and axial PD MRI images. These images demonstrate the axial variations of the distal radioulnar joint and the extensor carpi ulnaris (ECU) tendon during forearm pronation (top row), neutral position (middle row), and supination (bottom row). These modifications are observable on MRI. Notably, the radial portion of the radioulnar ligaments, which surrounds the articular disc, remains stable and is not affected by movement. Pronation- and supination-related changes occur primarily at the ulnar insertion of the TFCC. The ulnar portion of the radioulnar  ligaments, , varies according to pronation, supination, and neutral positions

Positioning the patient in the “Superman” pose, with the arm raised above the head, aligns the TFCC with the isocenter and optimizes image quality. The forearm should ideally be in a neutral position; pronation or supination alters ulnar insertions and decreases sensitivity and specificity for certain lesions, whereas the morphology of the articular disc and radial insertions remains unchanged (Fig. 7) [59, 60].

Three-dimensional (3D) MRI acquisitions with isotropic voxels allow multiplanar reconstructions from a single acquisition, reduce partial volume artifacts, and shorten overall scan time. Dixon-based fat suppression provides improved homogeneity in small field-of-view imaging [58]. Fluid-sensitive sequences identify tears as fluid discontinuities, whereas proton-density sequences detect intradiscal degeneration [53, 61]. Radial acquisitions, such as PROPELLER or BLADE, reduce motion and vascular pulsation artifacts, particularly on axial slices [58].

MR arthrography under fluoroscopic guidance shows higher sensitivity and specificity than conventional MRI for TFCC lesions. It uses gadolinium to enhance contrast, highlighting complete tears through extravasation, an indirect sign of fibrocartilage or insertional disruption [53]. Multiple meta-analyses and recent studies have confirmed the diagnostic superiority of MR arthrography, particularly for peripheral lesions [62–66] At 3 T, MR arthrography achieves high sensitivity and specificity for both central and peripheral TFCC lesions (93.3% and 97%, respectively) [67]. Suspicion of foveal tear represents the primary indication, especially when conventional MRI is inconclusive [68].

MR arthrography relies on a dual-injection technique. The first injection is performed in the DRUJ with 0.5 mL of iodinated contrast (iopamidol 300 mg I/mL) and 0.5 mL of gadolinium (4 mmol/L, Magnevist, Schering). The total volume may reach 4 mL if contrast migrates into the radiocarpal joint. If no passage occurs, a second injection is performed in the midcarpal joint. An additional radiocarpal injection primarily evaluates cartilage and is not required for the TFCC.

Lesion detection depends on contrast passage, indicative of fibrocartilage or insertional disruption. Passage limited to the foveal insertion indicates an isolated foveal tear, whereas passage into the radiocarpal joint reflects involvement of the fovea and ulnar styloid, radial insertions, or a central tear.

T1-weighted turbo spin-echo (TSE) sequences assess osseous structures, useful for small avulsion fractures, while proton-density sequences detect bone marrow edema and soft tissue changes. A SPIR sequence allows visualization of the contrast agent. Table 1 summarizes our protocol.

**Table 1:** Sequence parameters on 3-T MRI

3-T MRI							3-T MR arthrography: all previous sequences +
Sequence	T1 TSE tra	T1 TSE	3D PD SPAIR	PD SPAIR	PD SPAIR		SPIR
Imaging plane	Transverse	Coronal	Coronal	Transverse	Coronal	Sagittal	Coronal
Sequence type	2D TSE	2D TSE	2D TSE	2D TSE	2D TSE	2D TSE	2D TSE
TR (ms)	566	546	1400	2282	2321	2002	628
TE (ms)	9,8	15	32	30	30	30	11
FOV (mm)	80 × 100	100 × 100	110 × 110	80 × 90	100 × 110	80 × 89	100 × 100
Matrix	268 × 245	500 × 396	316 × 268	268 × 248	252 × 243	200 × 181	332 × 262
Voxel size (mm) x (mm)	0.30 × 0.41	0.20 × 0.25	0.35 × 0.41	0.30 × 0.36	0.40 × 0.45	0.40 × 0.49	0.30 × 0.38
Silice thickness (mm)	2.5	2.5	0.45	2.5	2	2	2
Interslice gap (mm)	0.25	0.25	0	0	0.2	0	0.2
Number of aqcuisitions	2	1	2	2	1	1	1
Parallel imaging technique	CS	CS	CS	AI-CS	AI-CS	SENSE	CS
Acceleration factor	2	2	6	2.8	2	1.2	1.3
Phase encoding direction	RL	RL	RL	RL	FH	FH	RL
In plane resolution (mm2)	0.16 × 0.16	0.18 × 0.18	0.2 × 0.2	0.18 × 0.18	0.25 × 0.25	0.23 × 0.23	0.18 × 0.18
Aqcuisition time	1 min 39	1 min 31	5 min 33	2 min 12	2 min 14	2 min 44	2 min 20

*CS* compressed sensing, *AI-CS* compressed sensing with deep learning reconstrution

### Normal MRI appearance of the TFCC

**Fig. 8:**
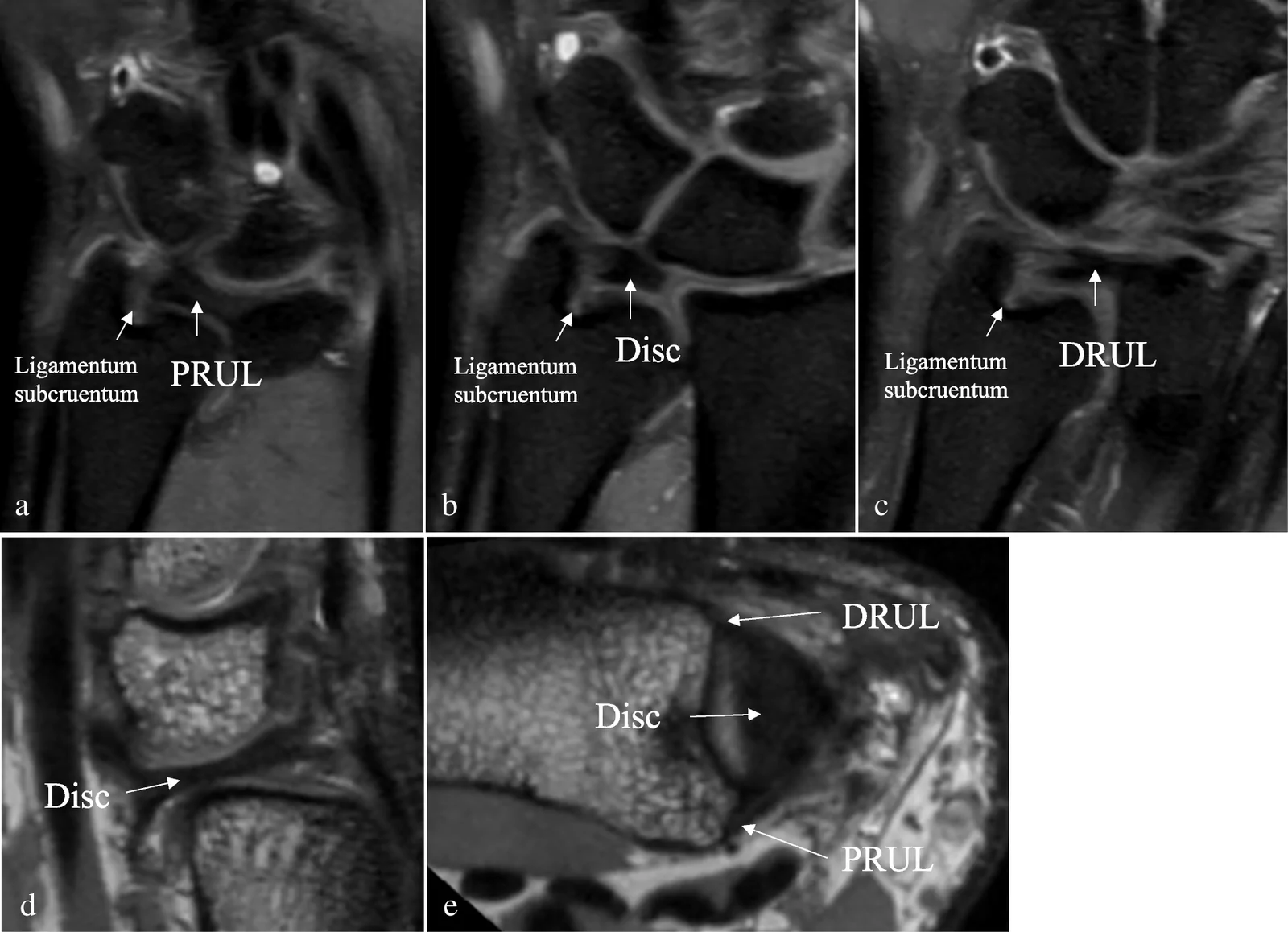
Normal appearance of the triangular fibrocartilage complex on MRI. **a**–**c** Coronal plane; **d** sagittal plane; **e** axial plane. PD FS MRI images (**a**–**c**) and standard PD MRI images (**d**, **e**) demonstrate the articular disc of the TFCC, its radial and ulnar attachments, as well as the ligamentum subcruentum. The TFCC appears hypointense on all sequences, whereas the ligamentum subcruentum shows intermediate signal intensity. In the coronal plane (**a**–**c**), the TFCC has an elongated triangular shape, with its apex attached to the radial sigmoid notch and its base connecting the foveal and ulnar styloid insertions. In the sagittal plane (**d**), it is visualized as a biconcave disc resting on the ulnar head. In the axial plane **(e**), it displays a semi-circular morphology above the radial sigmoid notch, with peripheral insertions. The palmar (PRUL) and dorsal (DRUL) radioulnar ligaments are hypointense

The triangular fibrocartilage, along with its radial and ulnar insertions (foveal and ulnar styloid), exhibits low signal intensity on all FSE and proton-density sequences. In contrast, the ligamentum subcruentum demonstrates a slightly higher intermediate signal intensity (Fig. 8).

In the coronal plane, the triangular fibrocartilage appears as an elongated triangle, with its apex attached to the radial sigmoid notch and its base connecting the foveal and ulnar styloid insertions. In the sagittal plane, it is visualized as a biconcave disc resting on the ulnar head, framed by the palmar and dorsal radioulnar ligaments (Fig. 8). In the axial plane, it assumes a semicircular shape over the radial sigmoid notch, with peripheral insertions at the fovea and ulnar styloid (Fig. 8).

The palmar ulnotriquetral ligament and the ulnolunate ligament appear hypointense and may show heterogeneous signal. Together, they form a trapezoidal configuration (Fig. 5). These ligaments, broad but thin in the coronal plane, are best appreciated on sagittal images. However, with high-quality imaging, they can also be visualized in the coronal (Fig. 5) and axial planes, with the ulnotriquetral ligament generally more readily identifiable than the ulnolunate ligament [58].

The dorsal ulnotriquetral ligament appears hypointense on all sequences. It shares a broad insertion on the dorsal ridge of the triquetrum with the dorsal radiotriquetral ligament. The dorsal ulnotriquetral ligament is best visualized on sagittal slices but can also be evaluated in coronal and axial planes (Fig. 5).

The ECU tendon has a proximal sheath that inserts on the dorsal and ulnar surfaces of the ulnar styloid, the fovea, and the DRUL. This insertional region is indistinguishable on MRI and appears markedly hypointense on all sequences, best appreciated on axial slices. Distally, the sheath inserts on the dorsal aspect of the triquetrum.

The various components of the MH are visible on MRI. Its ulnar styloid component is hyperintense on T1-weighted images and hypointense on PDFS sequences (reflecting the fatty portion), whereas the radial portion (ligamentous) shows low signal on both T1 and PDFS. The radioulnar, collateral, and distal components generally demonstrate low signal intensity on both T1 and PDFS sequences.

### MRI variants and diagnostic pitfalls

Beyond the morphological variations described in the anatomical section, several imaging appearances of the TFCC may mimic pathology on MRI. Recognition of these imaging variants and pitfalls is essential to avoid diagnostic errors.

#### Ligamentum subcruentum

The ligamentum subcruentum exhibits intermediate-to-high signal intensity on proton density and FSE sequences [58, 69]. Its shape can vary from a thin band to a broader, more triangular configuration. Misinterpretation of this signal or shape as a peripheral triangular fibrocartilage tear is a common pitfall, particularly on axial and coronal planes (Fig. 8). A normal TFCC does not demonstrate enhancement after contrast administration; however, enhancement may occasionally be observed at the site of a peripheral tear or rupture. Careful evaluation allows the mild enhancement often seen in the ligamentum subcruentum to be distinguished from a true pathological lesion, preventing false-positive interpretations [58].

#### Articular disc–cartilage interface

The insertion of the triangular fibrocartilage into the hyaline cartilage of the sigmoid notch of the radius normally demonstrates intermediate signal intensity and should not be mistaken for a tear at this insertion (Fig. 4h, i) [69].

#### Prestyloid and ulnocarpal recesses

Synovial outpouchings such as the prestyloid recess frequently exhibit fluid signal and may extend radially or ulnarly, potentially appearing as a cleft within the disc (Fig. 6e) [69]. Recognition of these recesses prevents misdiagnosis of small central or peripheral tears.

#### Distal radioulnar ligament variability

The DRUL and PRUL are normally hypointense but may show a striated appearance, particularly at their ulnar attachments (Fig. 3g, h) [70, 71]. These ligaments may also be thicker, partially bifid, or exhibit variable signal along their course. The foveal and styloid attachments of the radioulnar ligaments can change orientation and signal intensity with forearm rotation. In extreme pronation or supination, apparent elongation, partial detachment, or altered fiber orientation may be visualized, representing normal kinematic variation [59] rather than injury. These positional variations can lead to false-positive interpretations (Fig. 7). Following intravenous gadolinium administration, it is important to distinguish the normal synovial enhancement at the foveal reflection from a tear of the foveal attachment. This distinction is essential to avoid confusing physiologic enhancement with a true pathological lesion on MRI evaluation of the TFCC [58]. The most common source of misinterpretation on TFCC MRI is the presence of normal synovial tissue along the ulnar aspect of the wrist, which can make assessing the integrity of the foveal attachment particularly challenging. Assessment of a partial foveal tear, or even a complete but non-retracted tear, can be challenging due to the presence of synovitis or tissues located within the prestyloid recess. Furthermore, partially healed or partial foveal tears may exhibit intermediate signal intensity, potentially mimicking a complete tear [58]. A repaired or healed foveal tear may also demonstrate increased T2 signal on MRI, simulating a tear [72].

**Fig. 9:**
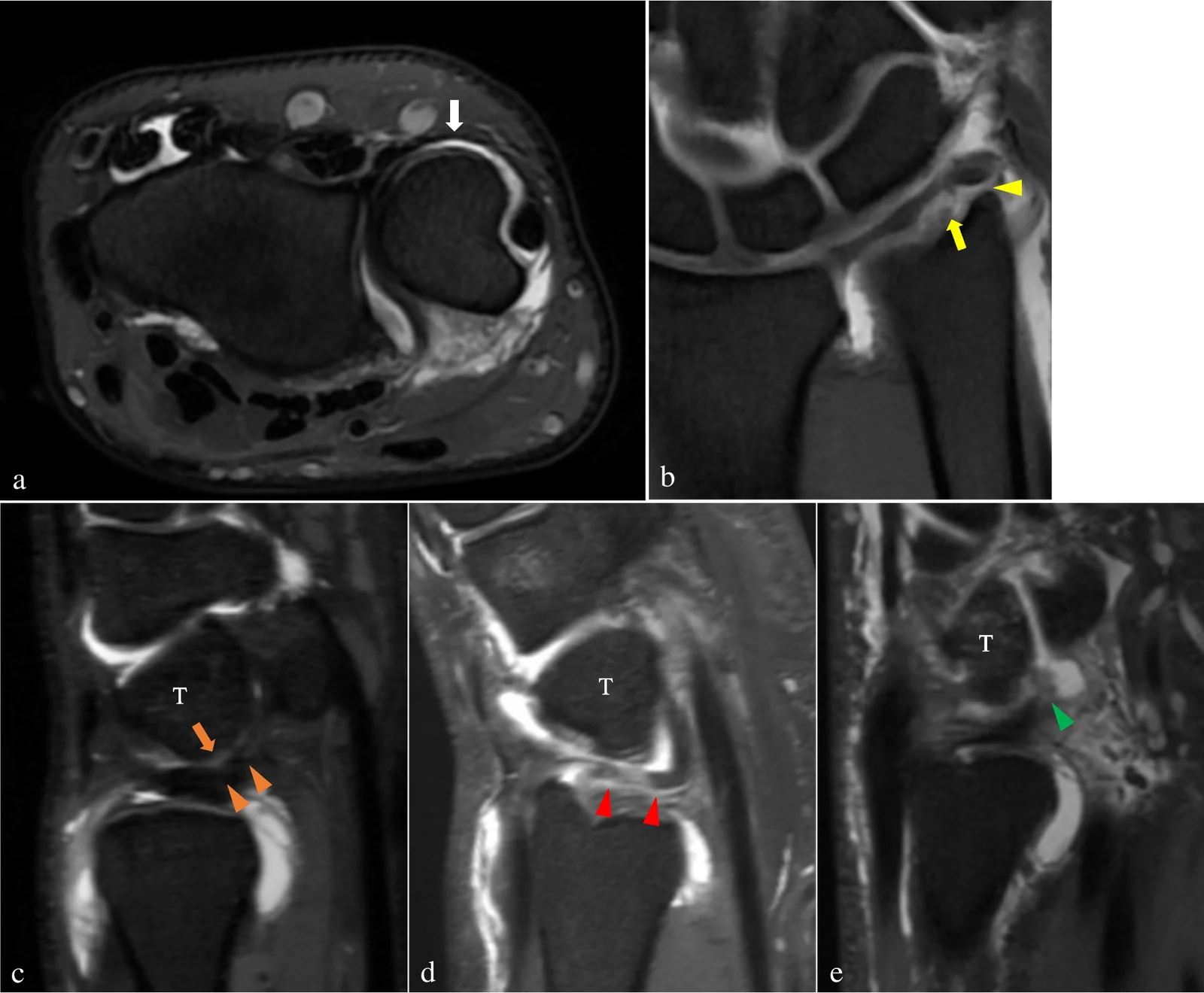
Dorsal DRUJ subluxation associated with foveal TFCC tear and ulnar styloid pseudarthrosis on MR arthrography (**a**, **b**): **a** Axial PD FS MRI image showing dorsal subluxation of the ulna relative to the sigmoid notch of the radius (white arrow). This indirect sign may be misinterpreted as a foveal TFCC tear, representing a false positive if the fovea itself is not carefully assessed. **b** Coronal T1 FS MR arthrography image demonstrating true detachment of the foveal TFCC insertion (yellow arrow) and ulnar styloid pseudarthrosis (yellow arrowhead). Contrast passage into the radiocarpal joint confirms articular communication. A true foveal tear may be overlooked if not specifically sought, representing a false negative. Key point: In the presence of dorsal DRUJ subluxation, systematic evaluation for TFCC tears, particularly foveal, is essential to avoid diagnostic errors. Deep palmar ulnotriquetral ligament bursa on MR arthrography (**c**): **c** Sagittal PD FS MR arthrography image showing a small bursa (orange arrow) located deep to the palmar insertion of the ulnotriquetral ligament on the triquetrum (orange arrowheads). This structure can mimic a partial ligament tear if not correctly identified, resulting in a false positive. Careful assessment of margins, precise location, and absence of ligament disruption allows differentiation of this normal anatomical bursa from a true tear. Key point: Recognition of this anatomical bursa is crucial to avoid false positives when interpreting TFCC MRIs, thereby improving diagnostic accuracy and preventing unnecessary interventions. Longitudinal (“split”) tear of the palmar ulnotriquetral ligament and triquetral insertion lesion on sagittal PD FS MRI images (**d**, **e**):**d** Sagittal PD FS MRI image showing an isolated longitudinal (split) tear of the palmar ulnotriquetral ligament (red arrowheads). This lesion can be subtle and easily overlooked, resulting in false negatives. Careful attention is required to avoid confusing it with a normal ligament or anatomical variant. **e** Illustrative schematic of the triquetral insertion lesion of the palmar ulnotriquetral ligament (green arrowhead), highlighting the distal attachment that may be missed on MRI, potentially leading to false negatives, and its clinical importance for distal radioulnar and ulno-carpal joint stability. Key point: Awareness of these longitudinal tears and systematic evaluation of the triquetral insertion are essential to prevent missed diagnoses and to maintain distal radioulnar and ulno-carpal joint stability. T, triquetrum

Although dorsal displacement of the DRUJ on MR imaging does not definitively indicate a tear of the fovea or other components of the TFCC, it serves as a valuable sign that prompts the radiologist to examine the ulnar wrist structures more carefully (Fig. 9a, b) [58, 73]. Even in cases where clinical and MR evidence of instability is present, the foveal attachment may remain intact, albeit lax and functionally insufficient [58, 74].

#### Palmar ulnocarpal ligaments

On MRI, the palmar ulnotriquetral ligament and ulnolunate ligament may exhibit heterogeneous signal intensity and variable thickness (Fig. 5). Occasionally, accessory fibers of these ligaments can be visualized, which may mimic partial tears.

A small bursa lies deep at the insertion of the palmar ulnotriquetral ligament on the triquetrum. Although this bursa may be mistaken for a partial tear, careful image analysis allows differentiation from a true pathological lesion (Fig. 9c) [58]. Longitudinal (“split”) tears of the palmar ulnotriquetral ligament, first described by Tay et al. [75], are often associated with a single traumatic event, such as a fall on an outstretched hand (Fig. 9d) [58]. Ringler et al. (2013) reported that non-contrast 3 T MRI has low sensitivity for detecting these longitudinal tears (30–58% depending on the reader), with moderate specificity (60%), indicating that MRI alone remains limited for definitive diagnosis (Fig. 9e) [76]. Awareness of these lesions and their systematic evaluation on MRI is important, as failure to recognize them may lead to DRUJ instability and ulno-carpal instability.

#### Dorsal ulnotriquetral ligament

**Fig. 10:**
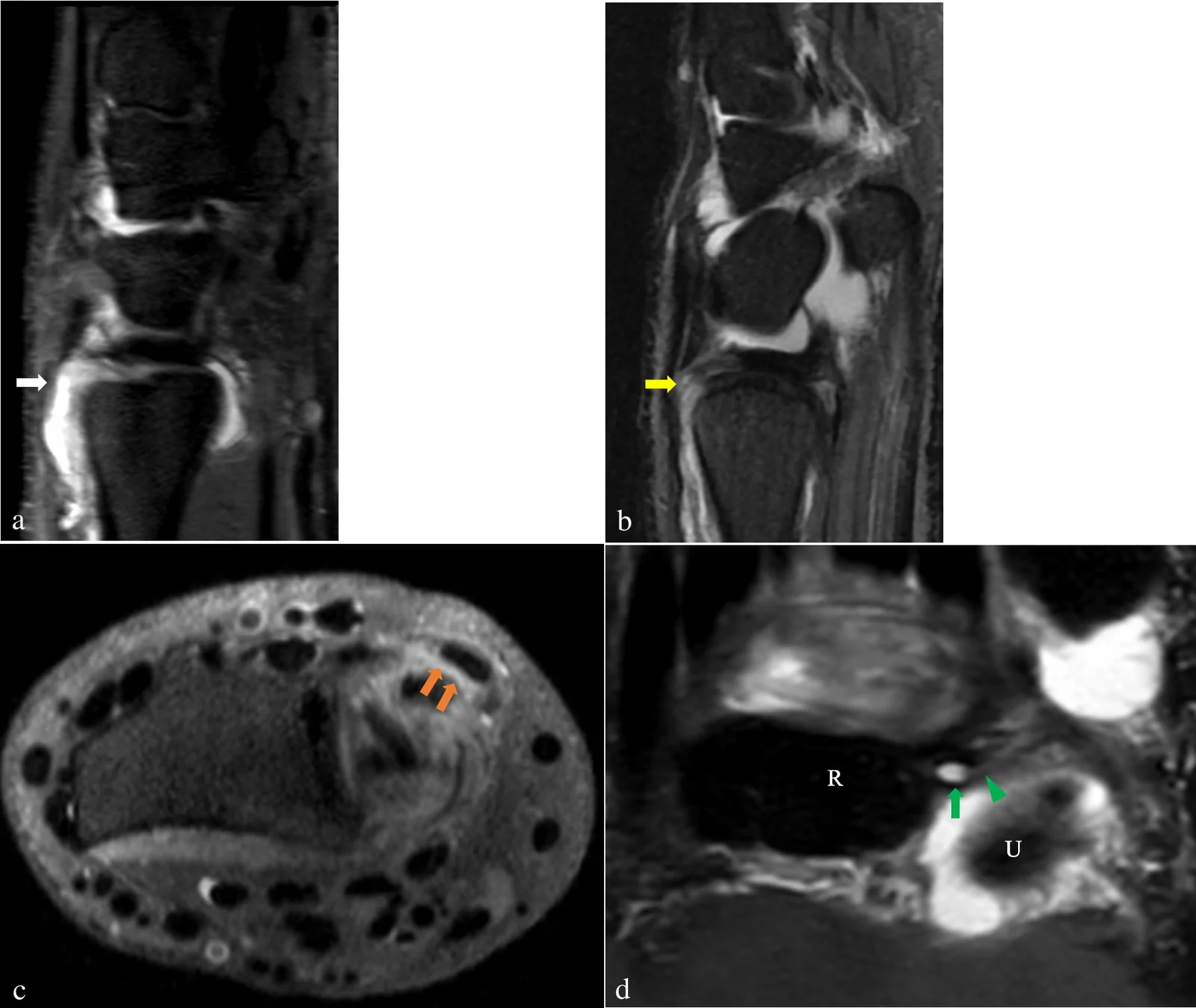
Dorsal ulnotriquetral ligament tear and post-injury synovitis (**a**, **b**): **a** Sagittal PD FS MRI image showing a tear of the dorsal ulnotriquetral ligament (white arrow). **b** Sagittal PD FS MRI image showing an appearance suggestive of a dorsal ulnotriquetral ligament tear (yellow arrow). However, arthroscopy demonstrated an intact ligament with interposed synovial tissue and post-injury synovitis affecting the dorsal ulnotriquetral ligament from a previous lesion. Ligamentous and periligamentous hyperintensity may mimic an active tear and lead to false-positive interpretations; MRI differentiation relies on careful evaluation of ligament continuity and correlation with the clinical context. Key point: Dorsal ulnotriquetral ligament lesions require careful analysis of sagittal images. False negatives: lack of recognition may cause persistent pain. Post-injury synovial interposition and synovitis can simulate an active tear, representing a potential source of false positives. Secondary sheath detachment of the ECU tendon and tendon instability (**c**):**c** Axial PD FS MRI image showing fluid signal along the secondary ECU tendon sheath, with detachment at its insertions on the TFCC (orange arrows). This signal may be mistaken for simple sheath fluid if the extension into the TFCC is not carefully evaluated. Key point: Secondary ECU sheath detachment requires careful assessment of the fluid signal. Unawareness of this fluid extension into the TFCC can result in false-negative interpretation. Arthrosynovial cyst adjacent to the radial insertion of the palmar radioulnar ligament (**d**): **d** Coronal PD FS MRI image showing a small cyst (green arrow) adjacent to the radial insertion of the palmar radioulnar ligament. The ovoid shape, homogeneous fluid signal, and the presence of an articular pedicle (green arrowhead) allow differentiation of this structure from a true ligament tear. Key point: Knowledge of the topography and imaging features of arthrosynovial cysts is essential to avoid false-positive MRI interpretations

Lesions of the dorsal ulnotriquetral ligament (Fig. 10a) [12, 77, 78] most commonly present with ulnar-sided wrist pain while generally causing little or no instability of the DRUJ. There is a risk of false-negative findings when this lesion is not recognized, which may result in persistent symptoms.

Interposition of synovial tissue and post-lesional synovitis can mimic an active ligament rupture on MRI, representing a common source of false-positive findings (Fig. 10b). Careful evaluation of sagittal images, together with clinical correlation, is essential to distinguish a true rupture from an old lesion or isolated ligamentous hyperintensity.

#### Meniscus homologue

The MH exhibits a heterogeneous signal. Awareness of its components helps prevent misinterpretation as tears or degeneration (Fig. 6).

#### ECU tendon subsheath

Detachment of the ECU tendon subsheath may be mistaken for fluid within the sheath. A fluid signal extending along the subsheath and its attachments to the TFCC indicates a rupture of the subsheath (Fig. 10c), which can lead to ECU instability.

#### Ulnar collateral ligament of the wrist

The ulnar collateral ligament may not be readily distinguished as a separate structure from the MH and the insertion of the ECU tendon subsheath [58].

#### Arthrosynovial ganglia of the ulnar wrist

Ganglia may be mistaken for TFCC lesions. They are typically of arthrosynovial origin and can be identified by their ovoid or rounded shape, fluid-like signal, and connection to the joint via a stalk or pedicle (Fig. 10d). These ganglia may occasionally be associated with central tears or perforations of the TFCC, but they generally do not arise from the TFCC itself [58]. Correct recognition of these ganglia is essential to avoid misdiagnosis and false-positive interpretations on MRI.

#### Displaced TFCC flap tears

Displaced flap tears of the TFCC represent an uncommon but clinically relevant pattern of TFCC injury that may be overlooked on routine imaging. In a large retrospective series of 3411 wrist MRI examinations, Boutin and Fritz reported a frequency of approximately 0.5% for displaced TFCC tears, corresponding to roughly one case in every 200 wrist MRI studies [79]. These lesions are characterized by a partial- or full-thickness defect of the TFCC associated with a displaced fibrocartilaginous fragment, which typically remains connected to the parent TFCC by a narrow pedicle. On MRI, this pedunculated configuration produces a characteristic appearance described as the “comma sign,” reflecting the curved morphology of the residual attachment between the displaced fragment and the torn TFCC.

In most reported cases, the displaced fragment migrates proximally into the DRUJ, most frequently within the volar recess. This displacement may produce mechanical symptoms or restriction of forearm rotation, particularly limitation of supination, due to interposition of fibrocartilaginous tissue within the joint space. Recognition of this entity is clinically important because displaced TFCC flaps may not be reliably visualized during standard radiocarpal arthroscopy, which does not systematically explore the DRUJ. Consequently, preoperative MRI identification of a displaced flap may provide critical information for surgical planning and may prompt dedicated DRUJ arthroscopic assessment.

From an imaging perspective, the combination of a TFCC defect, an adjacent low-signal fragment, and a pedunculated connection forming the comma sign should raise suspicion for a displaced TFCC flap tear. Awareness of this pattern helps differentiate it from small loose bodies, degenerative fragments, or imaging artifacts and underscores the importance of careful evaluation of the DRUJ recesses on multiplanar MRI sequences.

#### Ulnar-sided contrast extravasation in MR arthrography

Ulnar-sided contrast extravasation during wrist MR arthrography is a common finding that can occur even in the absence of a true peripheral TFCC tear. Gietzen et al. (2022) demonstrated that anatomical variations of the prestyloid recess may mimic ulnar leakage, highlighting the importance of recognizing these variants to avoid false-positive interpretations [80]. Similarly, Lee et al. (2019) reported that extravasation along the ulnar aspect of the wrist occurred in approximately 57% of radiocarpal joint arthrograms and was not consistently associated with clinical or arthroscopic evidence of TFCC injury [81]. These findings indicate that ulnar contrast leakage may result from procedural factors or normal anatomical variation rather than a true peripheral tear, and careful evaluation of multiplanar MR sequences is recommended to distinguish between iatrogenic or variant-related leakage and genuine TFCC disruption.

#### Clinical correlation of TFCC MRI findings

Interpretation of TFCC abnormalities on MRI should always consider the patient’s clinical context. The prevalence of TFCC abnormalities increases with age, and incidental findings unrelated to symptoms are relatively common. A meta-analysis of seven studies demonstrated that TFCC abnormalities can occur at any age, with some variations related to age or high-impact sports participation, but no specific tear pattern reached statistical significance [82].

In another study, TFCC abnormalities were reported on MRI in 44% of patients without ulnar-sided wrist pain. Their prevalence increased with age, rising from 28% in patients under 40 years to 67% in those over 40 years [83]. These findings highlight the relatively high rate of signal alterations observed on MRI even in asymptomatic individuals.

In adolescents (mean age 15 years), MRI showed relatively low sensitivity for detecting TFCC lesions compared with arthroscopy (14–71%), although specificity remained high (82–94%) with an overall diagnostic accuracy of 70–83%. Approximately 85% of patients reported a prior traumatic event, suggesting that MRI may underdiagnose TFCC abnormalities in this younger population [84, 85].

Finally, there is evidence that observer experience affects diagnostic accuracy, with the expertise of the radiologist or surgeon playing an important role in the reliability of MRI interpretation for TFCC lesions [86].

## Conclusion

The TFCC functions as an integrated anatomical and biomechanical unit and represents a key stabilizer of the ulnar side of the wrist, linking the DRUJ and the ulnocarpal joint. Recent advances in arthroscopic anatomy support a three-dimensional, tripartite conceptualization of the TFCC, composed of the articular disc, the distal radioulnar ligaments providing primary stability, and a continuous peripheral capsular structure encompassing the palmar and dorsal ulnocarpal ligaments, the MH, and the ECU tendon sheath. This organization ensures coordinated stabilization during pronation and supination.

MRI, particularly three-dimensional isotropic sequences and MR arthrography, allows precise evaluation of the ligamentous and fibrocartilaginous components, facilitating reliable diagnosis and surgical planning. Awareness of normal anatomical variants and dynamic changes related to forearm position is essential to distinguish pathological lesions from normal structures and to avoid diagnostic pitfalls. A comprehensive understanding of the updated anatomy, biomechanics, and imaging characteristics of the TFCC optimizes the assessment and management of ulnar-sided wrist injuries and contributes to improved clinical outcomes.

## Data Availability

Not applicable.

## Abbreviations

DRUJ: Distal radioulnar joint
DRUL: Dorsal distal radioulnar ligament
ECU: Extensor carpi ulnaris
MRI: Magnetic resonance imaging
MH: Meniscus homologue
PRUL: Palmar distal radioulnar ligament
TFCC: Triangular fibrocartilage complex
